# Aligning Cross-Species Interactomes for Studying Complex and Chronic Diseases

**DOI:** 10.3390/life13071520

**Published:** 2023-07-06

**Authors:** Marianna Milano, Pietro Cinaglia, Pietro Hiram Guzzi, Mario Cannataro

**Affiliations:** 1 Department of Experimental and Clinical Medicine, University Magna Græcia, 88100 Catanzaro, Italy; 2 Data Analytics Research Center, University Magna Græcia, 88100 Catanzaro, Italy; cinaglia@unicz.it (P.C.); hguzzi@unicz.it (P.H.G.); cannataro@unicz.it (M.C.); 3 Department of Health Sciences, University Magna Græcia, 88100 Catanzaro, Italy; 4 Department of Medical and Surgical Sciences, University Magna Græcia, 88100 Catanzaro, Italy

**Keywords:** Alzheimer’s disease, Parkinson’s disease, PPI network, network alignment, local network alignment

## Abstract

Neurodegenerative diseases (NDs) are a group of complex disorders characterized by the progressive degeneration and dysfunction of neurons in the central nervous system. NDs encompass many conditions, including Alzheimer’s disease and Parkinson’s disease. Alzheimer’s disease (AD) is a complex disease affecting almost forty million people worldwide. AD is characterized by a progressive decline of cognitive functions related to the loss of connections between nerve cells caused by the prevalence of extracellular Aβ plaques and intracellular neurofibrillary tangles plaques. Parkinson’s disease (PD) is a neurodegenerative disorder that primarily affects the movement of an individual. The exact cause of Parkinson’s disease is not fully understood, but it is believed to involve a combination of genetic and environmental factors. Some cases of PD are linked to mutations in the LRRK2, PARKIN and other genes, which are associated with familial forms of the disease. Different research studies have applied the Protein Protein Interaction (PPI) networks to understand different aspects of disease progression. For instance, Caenorhabditis elegans is widely used as a model organism for the study of AD due to roughly 38% of its genes having a *human* ortholog. This study’s goal consists of comparing PPI network of *C. elegans* and *human* by applying computational techniques, widely used for the analysis of PPI networks between species, such as Local Network Alignment (LNA). For this aim, we used L-HetNetAligner algorithm to build a local alignment among two PPI networks, i.e., *C. elegans* and *human* PPI networks associated with AD and PD built-in silicon. The results show that L-HetNetAligner can find local alignments representing functionally related subregions. In conclusion, since local alignment enables the extraction of functionally related modules, the method can be used to study complex disease progression.

## 1. Introduction

Neurodegenerative diseases (NDs) are complex disorders characterized by progressive damage and loss of neurons in the central nervous system (CNS) leading to mobility and/or cognition impairments. For instance, Alzheimer’s disease (AD) is a progressive neurodegenerative disorder that affects the brain, primarily causing problems with memory, thinking, and behavior. AD is the most common cause of dementia, a general term for a decline in cognitive abilities severe enough to interfere with daily life [1,2]. The pathogenesis of AD is related to be a complex interplay of genetic, environmental, and lifestyle factors [3]. The disease is characterized by the accumulation of abnormal protein deposits, including beta-amyloid plaques [4] and TAU tangles [5] in the brain. The accumulation causes a loss of connections between nerve cells and the eventual death of these cells, resulting in the progressive decline of cognitive functions.

The symptoms of AD usually develop slowly and worsen over time. Early signs may include forgetfulness, difficulty with language and problem-solving, confusion, disorientation, and mood and behavior changes. As the disease progresses, individuals may experience significant memory loss, impaired judgment, and difficulties with daily tasks. In the later stages, people with AD may lose the ability to converse or respond to their environment [6].

Parkinson’s disease [7] (PD) is a chronic and progressive neurodegenerative disorder that primarily affects the movement of individuals. It is the second most common neurodegenerative disease after AD. PD is characterized by motor symptoms such as tremors, rigidity, and bradykinesia [8]. A combination of genetic and environmental factors causes it. Some cases of PD are associated with mutations of the (PARK2) gene [9].

While there is currently no cure for AD and PD, various treatments and interventions can help manage symptoms and improve the quality of life for individuals with the condition [2]. These may include medication to temporarily improve cognitive function or manage behavioral symptoms and non-drug approaches such as cognitive stimulation, physical exercise, and support from caregivers [2,10,11].

Current studies focus on understanding the disease mechanism, i.e., ongoing research to uncover the molecular and cellular mechanisms involved in the development of complex disease [12]. The complete elucidation of these mechanisms may suggest biomarkers for the early detection of the disease and identification of potential drug targets.

Such studies require a model to represent association among genes and protein related to diseases in order to discover disrupted mechanisms related to diseases. Network sciences and in particular protein–protein interaction (PPI) networks may represent a useful framework to shed light on disease mechanisms [13,14,15]. PPI networks are networks whose nodes are proteins and edges among them represent their biochemical associations. Many studies based on PPI networks obtained significant results, discovering disease complexities at the protein and gene levels [16,17,18]. In particular, PPI networks have been used to transfer knowledge among different species, i.e., from models to complex organisms [19], if proteins are conserved among different species [20].

*C. elegans* is a widely used model organism in biological research due to its well-characterized and straightforward anatomy. Although *C. elegans* does not develop any neurodegenerative diseases because it lacks certain key features of the *human* brain, it has been used to study various neurodegenerative disorders, including AD and PD [21,22,23].

Several genes associated with AD in *humans* have orthologs in *C. elegans*. For example, the amyloid precursor protein (APP) gene, which plays a crucial role in forming amyloid plaques in Alzheimer’s disease, has an ortholog in *C. elegans* called apl-1. Similarly, ptl-1 protein is the ortholog of *human* TAU protein. Similarly, different PD genes have orthologues in *C. elegans*, for example parkin ortholog, PDR-1. Typically, the *C. elegans* PPI network for studying neurodegenerative disorders is built by combining experimental and computational methods. Once the PPI network is constructed, analysis can be conducted on the network topology, i.e., to identify some key proteins or modules involved in neurodegenerative disorders-related processes or by performing a comparison among networks of different organisms.

A computational technique widely used for comparative analysis of PPI networks between species is Network Alignment (NA) [24,25,26]. NA algorithms enable us to elucidate the conservation among the evolution of molecular machinery or, similarly, the conservation of protein complexes among species by discovering common subnetworks (obtained with the Local NA approach) or whole similarities (obtained with the Global NA approach).

In [27], Apostolakou et at. applied the Global NA method to Alzheimer-related PPI networks in *humans* and *C. elegans* to find common biological pathways conserved in two organisms involved in AD.

To the best of our knowledge, using Local NA methods for comparison and analysis of neurodegenerative disease PPI networks remains unexplored.

Starting from these considerations, in this work, we apply Local NA to analyze PPI networks to discover proteins related to neurodegenerative disease in model organisms, like *C. elegans*, and in *humans*.

For this goal, we build in silico *C. elegans* and *human* PPI networks associated with AD and PD, and we applied the L-HetNetAligner algorithm to build a local alignment among two PPI networks. To our knowledge, this is the first study that applies the local alignment to compare PPI networks to study neurodegenerative disease. The results show that the extracted modules, representing the local alignment, are functionally related and present a higher biological relevance than by chance. The rest of this paper is organized as follows. Section 2 discusses the theoretical background. Section 4 presents the approach and discusses the results. Finally, Section 6 concludes the paper.

## 2. Related Work

### 2.1. Network Comparison

Several algorithms and techniques exist that can be used to assess similarities or differences among networks [28]. For instance, Graph Edit Distance is used to find a minimum cost set of operations (node and edge deletions or insertions) to transform a graph into another [29]. A comparison of the topological parameters of the graphs (degree distribution, clustering coefficient, average path length) may provide some insights into the differences between networks [30]. Comparison of mesoscale parameters, such as spectra of the graphs, communities as well as network motifs, may reveal differences at different levels [31,32].

### 2.2. Network Alignment Algorithms

Network alignment (NA) is a computational technique widely used for comparative analysis of PPI networks between species to predict evolutionary conserved components or sub-structures at a system data level.

Considering the interaction networks, the mapping from a specie into another one resides on the definition of such mapping, i.e., how to state that two proteins of two different species can be associated [33]. The correct choice of the association is a key problem [34,35]. Usually, the associated proteins should be orthologs, mainly when the alignment is used to explain evolutionary relationships. The definition of orthologs is often a too-restrictive criterion, so most algorithms try to choose the correspondent proteins based on their sequence similarity and eventually introduce other biological considerations.

Thus, the network alignment is a common problem that requires searching for a node mapping that best fits one network into another [36]. The problem of graph alignment consists of the mapping between two or more graphs to maximize an associated cost function that represents the similarity among nodes or edges. Formally, let $G_{1}=\left\{V_{1},E_{1}\right\}$ and $G_{2}=\left\{V_{2},E_{2}\right\}$ two graphs, where $V_{1,2}$ are sets of nodes and $E_{1,2}$ are sets of edges, the **graph alignment problem** consists of finding an alignment function (or a mapping) $f:V_{1}\to V_{2}$ such that the similarity between mapped entities is maximized. Thus, the alignment problem relies on the *(sub)-graph isomorphism problem*, which is computationally NP-hard in some general formulations, and heuristic methods should resolve it. The similarity between the graphs is defined by a cost function, $Q(G_{1},G_{2},f)$, also known as the quality of the alignment. Q expresses the similarity among two input graphs on a specific alignment *f*, and the formulation of Q strongly influences the mapping strategy [37]. In the literature, there exist different network alignment algorithms that investigate approximate solutions. The network alignment algorithms can be categorized as local or global alignment.

Local network alignment (LNA) algorithms search multiple small subnetworks with high similarity among input networks by producing a many-to-many node mapping. These subnetworks are conserved patterns of interaction that can correspond to conserved motifs or patterns of activities. LNA algorithms employ a two-step schema to build the alignment. At first, starting from a list of seed nodes selected from biological considerations, the algorithms integrate all the information in an auxiliary structure, usually called an alignment graph. Then they mined the graph for exciting evidence regions. The literature contains many network alignment algorithms to compare classical networks, also called homogeneous, due to the nodes representing a single entity. Otherwise, the network alignment algorithms are developed for specific networks, such as heterogeneous, i.e., networks where the nodes can represent different entities.

For example, L-HetNetAligner [38] is a local network alignment algorithm for comparing heterogeneous networks. L-HetNetAligner takes two heterogeneous networks and a set of similarities between node pairs as the input. Then, L-HetNetAligner creates the nodes of the alignment graph. Each node of the alignment graph represents a pair of nodes of input networks. The input similarity relationships guide the selection of node pairs. Therefore, each node is matched with the most similar node of the other network, and each node of the alignment graph represents a pair of similar nodes from the input networks. Once all nodes have been added to the graph, L-HetNetAligner creates the edges of the alignment graph. Edges are weighted according to the colors of corresponding nodes and topological considerations according to seven parameters: δ node distance related to the length of the shortest connecting path) *heterogeneous match* that set the weight of edges connecting the nodes in both input networks having the different colour, *homogeneous match* that set the weight of edges connecting the nodes in both input networks having the same color, *heterogeneous mismatch* that set the weight of edges connecting the nodes in a single network having the different color, *homogeneous match* that set the weight of edges connecting the nodes in a single network having the same color, *heterogeneous gap* that put the weight of edges connecting the adjacent nodes in a single network and they are at a distance lower than δ in the other network and all the nodes having the different color, *homogeneous gap* that set the weight of edges connecting the adjacent nodes in a single network and they are at a distance lower than δ in the other network and all the nodes having the same color. See [38] for complete details. The analysis of the input networks determines the presence of an edge in the alignment graph. Once the alignment graph is built, we use the Markov clustering algorithm (MCL) [39].

### 2.3. Semantic Similarity Measures

Biological knowledge can be defined via mathematical formulas that are useful to perform several tasks; for instance, we may use these for classification and comparison. In this context, a semantic similarity measure (i.e., SSM) is a function calculated between biological objects based, e.g., on their related terms. A similarity score is a metric defined over a set of features (e.g., terms), where the resulting distance is based on the likeness of their semantic meaning. For example, in a graph model consisting of genes (i.e., nodes) and their interactions (i.e., edges), we may calculate the similarity based on sub-structure matching or vector similarity. The latter denotes the similarity of two nodes measuring the distance between their associated vectors; the same approach may be mutually used for dissimilarity [40].

The Information Content (IC) method computes the similarity between two terms in according to their Most Informative Common Ancestor (MICA) terms, or Closest Common Ancestor (CCA) terms. We describe the following well-known IC-based measures: Resnik, Lin, and Jiang. The Resnik’s similarity measure [41] (i.e., $sim_{res}$) of two terms $T_{1}$ and $T_{2}$ of GO is based on the determination of the IC of their MICA.

(1) $$sim_{Res}=IC\left(MICA\left(T_{1},T_{2}\right)\right)$$

It mainly considers the common ancestor, and it does not take into account the distance among the compared terms and the shared ancestor.

Lin’s measure [42] (i.e., $sim_{Lin}$) assigns higher weights to more frequent categories in case of matches, and lower weights to less frequent ones in case of mismatches:

(2) $$sim_{Lin}=\frac{IC(MICA\left(T_{1},T_{2}\right)}{IC\left(T_{1}\right)+IC\left(T_{2}\right)}$$

Jiang’s measure [43] (i.e., $sim_{JC}$) takes into account the distance among terms by calculating the following formula:

(3) $$sim_{JC}=1-IC\left(T_{1}\right)+IC\left(T_{2}\right)-2*IC(MICA\left(T_{1},T_{2}\right)$$

## 3. Materials and Methods

### 3.1. Dataset: AD-Related PPI Networks

We queried the STRING [44] database to build the PPI network related to APP and TAU proteins (*human network* hereafter), and the PPI network related to apl-1 and ptl-1 (*C. elegans* network hereafter). We set the following parameters in the STRING database: (i) all interactions, (ii) score greater than 0.7. We obtain a *C. elegans* network, which contains 33 nodes and 82 edges, while the *human* network has 41 nodes and 157 edges. We used the OrthoList2 database [45] to find the *C. elegans* orthologs of *human* proteins.

### 3.2. Dataset: PD-Related PPI Networks

We queried the STRING [44] database to build the PPI network related to PARK-2 protein (*human network* hereafter), and the PPI network related to PDR-1 (*C. elegans* network hereafter). We obtain a *C. elegans* network, which contains 51 nodes and 438 edges, while the *human* network has 51 nodes and 651 edges. We used the OrthoList2 database [45] to find the *C. elegans* orthologs of *human* proteins.

### 3.3. Local Network Alignmnent

We built the local alignment among *C. elegans* network and *human* network AD-related and PD-related by applying the L-HetNetAligner algorithm. L-HetNetAligner takes as input the PPI networks and the orthologs proteins extracted as seed nodes. We set L-HetNetAligner parameters as follows: delta = 2, homogeneous match = 1, heterogeneous match = 0.9, homogeneous mismatch = 0.5, heterogeneous mismatch = 0.4, homogeneous gap = 0.2, heterogeneous gap = 0.1. Furthermore, we assigned a single color to the nodes of both input networks. We ran L-HetNetAligner on a Linux machine with Intel Core i5 and 4 GB of RAM and the algorithm built both local alignments in 60 s.

## 4. Results

We applied L-HetNetAligner algorithm to build the local alignment among *C. elegans* network and AD-related and PD-related networks.

For AD, the output consists of local alignment as six relevant modules. Table 1 reports the number of proteins belonging to each module.

For PD, the output consists of local alignment as five relevant modules. Table 2 reports the number of proteins belonging to each module.

To evaluate the quality of the local alignment, we consider the biological relevance of the extracted module in terms of functional similarity, which is a measure of functional relatedness. We used the semantic similarity concept applied to Gene Ontology vocabularies [46]. Therefore, we computed the semantic similarity values of the extracted modules using the three semantic similarity measures: Resnik’s with the Best-Match Average (BMA) approach [47], Lin [42] and Wang [48]. We also chose Wang’s measure because is a hybrid measure that computes the semantic similarity by considering the best path (in terms of weighted edges) among the gene products that outperforms other semantic similarities by producing results closer to *human* expectations (see [48] complete details). Thus, we computed for each module the semantic similarity score by applying Resnik’s, Lin’s and Wang’s measures.

Table 3 and Table 4 report the values of semantic similarity of each module computed with three measures for AD and PD.

Furthermore, to demonstrate that high and, therefore, significant values of semantic similarity are due to the functional relationship of the proteins that form the modules of the local alignment and not by chance, we compared our results with respect to randomly generated alignments.

**Figure 1:**
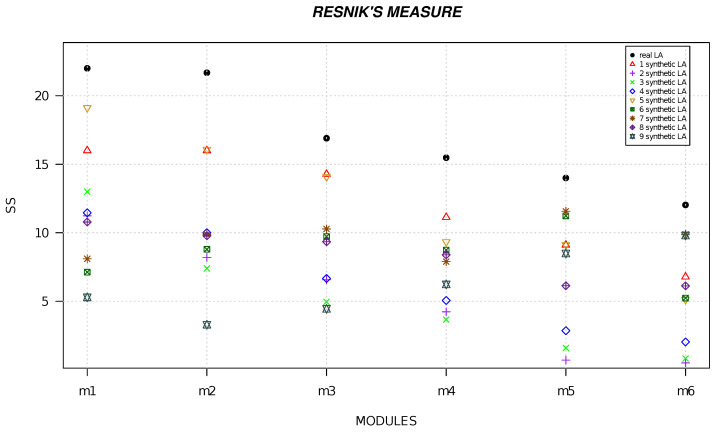
The figure shows the semantic similarity of six real modules (AD-related) (black marker) and synthetic modules (red, purple, green, blue, golden, dark green, orange, orchid, gray markers) computed with Resnik’s measure.

**Figure 2:**
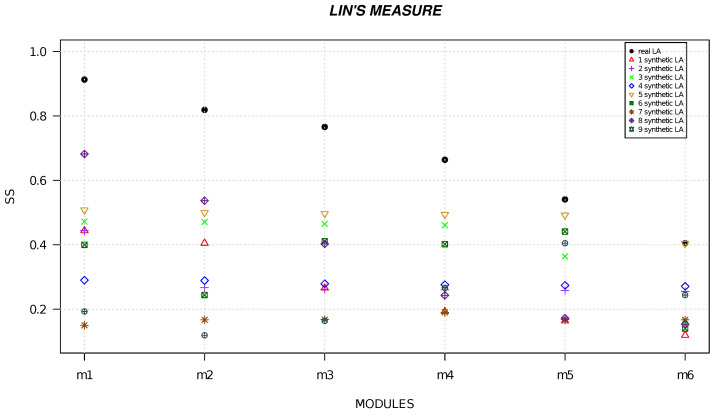
The figure shows the semantic similarity of real six modules (AD-related) (black marker) and synthetic modules (red, purple, green, blue, golden, dark green, orange, orchid, gray markers) computed with Lin’s measure.

**Figure 3:**
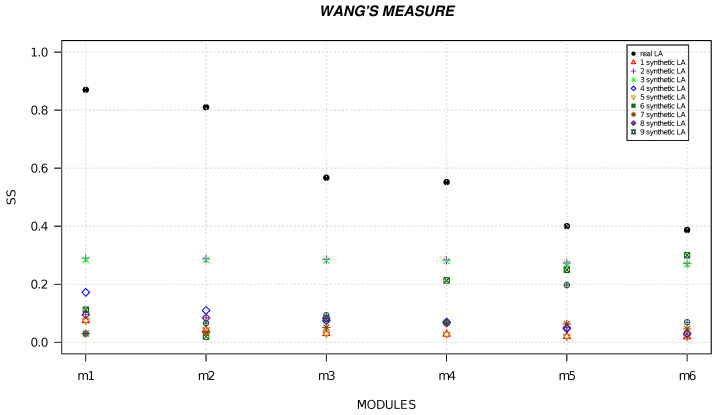
The figure shows the semantic similarity of real six modules (AD-related) (black marker) and synthetic modules (red, purple, green, blue, golden, dark green, orange, orchid, gray markers) computed with Wang’s measure.

**Figure 4:**
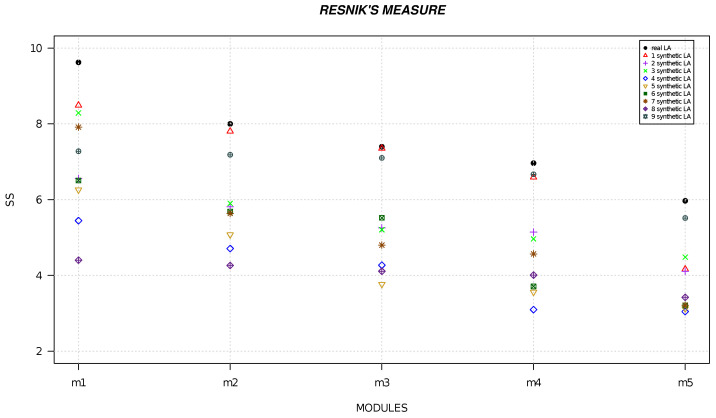
The figure shows the semantic similarity of real six modules (PD-related) (black marker) and synthetic modules (red, purple, green, blue, golden, dark green, orange, orchid, gray markers) computed with Resnik’s measure.

**Figure 5:**
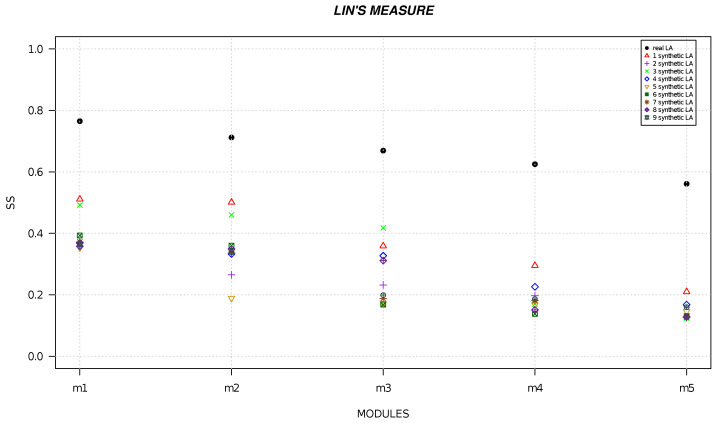
The figure shows the semantic similarity of real six modules (PD-related) (black marker) and synthetic modules (red, purple, green, blue, golden, dark green, orange, orchid, gray markers) computed with Lin’s measure.

**Figure 6:**
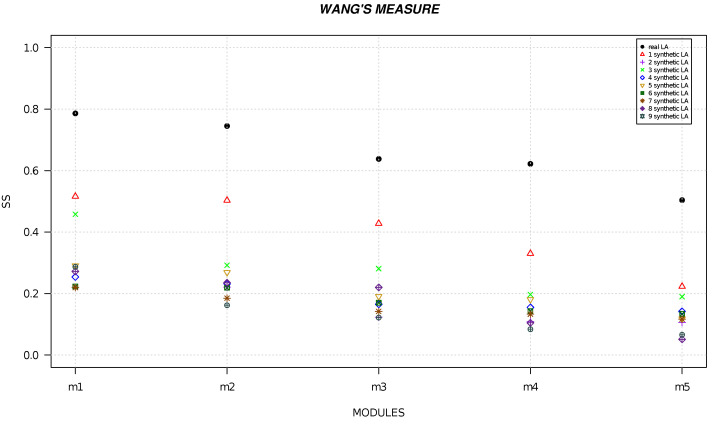
The figure shows the semantic similarity of real six modules (PD-related) (black marker) and synthetic modules (red, purple, green, blue, golden, dark green, orange, orchid, gray markers) computed with Wang’s measure.

We randomly generated six local alignments and five local alignments by using R software [49] and we calculated the semantic similarity for each alignment. Figure 1, Figure 2, Figure 3, Figure 4, Figure 5 and Figure 6 show the comparison of semantic similarity values of real local alignment and synthetic alignments. It is possible to note that for each module the semantic similarity values are greater in the case of real local alignment (report in the figure with the black marker). Furthermore, it is possible to find the same trend for each of the applied measures.

In conclusion, the real local alignment, i.e., the relevant modules, show a relatedness significantly higher than by chance.

## 5. Discussion

The study presented in the article explores the application of LNA to analyze PPI networks associated with AD and PD in model organisms such as *C. elegans* and *humans*. The goal of the study is to discover proteins related to AD and PD and investigate their functional relevance in both species.

The researchers constructed in silico PPI networks for *C. elegans* and *humans*, focusing on proteins associated with AD, such as APP and TAU in *humans* and their orthologs APL-1 and PTL-1 in *C. elegans*. Also, they constructed in silico PPI networks for *C. elegans* and *humans*, focusing on proteins associated with PD, such as PARK-2 and it ortholog PDR-1 in *C. elegans*. The L-HetNetAligner algorithm was then applied to perform local network alignment between the two PPI networks.

The results of the study demonstrate that the extracted modules are functionally related and possess a biological relevance higher than what would be expected by chance. This suggests that there are conserved biological pathways and interactions involved in AD and PD across these two species.

The findings of this study are significant for several reasons. Firstly, they highlight the potential of using network alignment techniques, specifically LNA, to compare PPI networks and gain insights into complex diseases such as AD and PD. By identifying conserved modules and interactions, researchers can uncover key proteins and pathways involved in the disease, leading to a better understanding of its underlying mechanisms.

Furthermore, the study demonstrates the utility of model organisms like *C. elegans* in studying neurodegenerative diseases such as AD and PD. Despite lacking certain features of the *human* brain and not developing AD and PD, *C. elegans* has orthologs of genes associated with AD and PD, making it a valuable tool for investigating aspects of the disease. The successful application of LNA to compare PPI networks in *C. elegans* and *humans* further supports the use of model organisms in disease research.

It is important to note that the study focused on local network alignment, which allows for the discovery of conserved functional network modules rather than a global comparison of entire interactomes. This approach enables the identification of specific protein complexes and interactions that are relevant to AD and PD, providing targeted insights into the disease processes.

Overall, the results of this study contribute to the growing field of network analysis in disease research. By combining computational techniques and biological knowledge, researchers can uncover conserved molecular mechanisms and pathways associated with AD and PD. This knowledge can potentially lead to the development of new therapeutic strategies and interventions for the treatment of AD and PD.

The use of *C. elegans* data to make inferences about humans has some drawbacks to consider. First, *C. elengans* has a relatively simple nervous system, while humans have a more complex nervous system with billions of neurons. Second, there are significant structural and functional differences between the two systems. Finally, humans also present differences among individuals due to environmental factors as well as genes and sex [50].

In conclusion, the study demonstrates the effectiveness of local network alignment in comparing PPI networks associated with AD and PD in *C. elegans* and *humans*. The findings highlight the importance of conserved interactions and pathways in the disease and provide valuable insights for further research and the development of targeted interventions.

## 6. Conclusions

AD is a complex neurodegenerative disorder due to the extracellular and intracellular accumulation of proteins, and its progression is still under study. Different research studies have applied the protein–protein interaction (PPI) networks to understand different aspects of disease progression. The aim of this study is to provide a network analysis approach to the study of AD. We built in silico the AD-related and PD-related *human* and the model organism *C. elegans* PPI networks. AD-related PPI networks were constructed for the *human* proteins APP and Tau and for their respective *C. elegans* orthologs APL-1 and PTL-1. PD-related PPI networks were constructed for the *human* protein PARK-2 and for its respective *C. elegans* ortholog PDR-1. Then, we compare the PPI networks by applying L-HetNetAligner. The results show that L-HetNetAligner is able to extract modules, representing the local alignment, that are functionally related and that present a biological relevance. In future works, we plan to extend the use of L-HetNetAligner to study different complex chronic diseases.

## Figures and Tables

**Table 1:** Local alignment consisting of six relevant modules built among *C. elegans* network and *human* network with L-HetNetAligner in AD. For each module, the table reports the number of proteins that form it.

Module	# Protein
1	28
2	28
3	12
4	12
5	2
6	2

**Table 2:** Local alignment consisting of six relevant modules built among *C. elegans* network and *human* network with L-HetNetAligner in PD. For each module, the table reports the number of proteins that form it.

Module	# Protein
1	50
2	30
3	35
4	21
5	15

**Table 3:** Semantic similarity values for each module forming the local alignment computed by applying Resnik’s, Lin’s and Wang’s measures for AD.

Local Alignment	Resnik	Lin	Wang
module 1	22	0.913	0.870
module 2	21.678	0.819	0.810
module 3	16.900	0.766	0.567
module 4	15.478	0.664	0.552
module 5	14.000	0.444	0.400
module 6	12.030	0.405	0.387

**Table 4:** Semantic similarity values for each module forming the local alignment computed by applying Resnik’s, Lin’s and Wang’s measures for PD.

Local Alignment	Resnik	Lin	Wang
module 1	9.624	0.765	0.786
module 2	8	0.712	0.745
module 3	7.398	0.669	0.638
module 4	6.592	0.625	0.622
module 5	5.97	0.561	0.504

## Abbreviations

The following abbreviations are used in this manuscript:

ND	Neurodegenerative diseases
AD	Alzheimer’s disease
PD	Parkinson’s disease
PPI	Protein-Protein Interaction
NA	Network Alignment
LNA	Local Network Alignment
GNA	Global Network Alignment
GO	Gene Ontology
SS	Semantic Similarity
